# Optimization of long-term graft survival after liver transplantation: the role of donor age

**DOI:** 10.1186/1471-2318-11-S1-A25

**Published:** 2011-08-24

**Authors:** Q Lai, F Melandro, G Spoletini, GB Levi Sandri, N Guglielmo, S Ginanni Corradini, PB Berloco, M Rossi

**Affiliations:** 1 Department of General Surgery and Organ Transplantation, Sapienza University, Rome, Italy

## Background

Nowadays, several solutions have been proposed for the minimization of both organ shortage and prolonged waiting times: the expansion of the donor pool using aged donors represents a possible solution [1]. However, it is not completely clear if the use of “extreme” donors could cause unacceptable post-transplant adjunctive risks [2]. Starting from these grounds, the aim of this study is to evaluate the impact of donor age on long-term graft survival.

## Materials and methods

From January 2001 to April 2009, 188 consecutive liver transplantations were performed at our Department. The entire cohort was stratified in 4 subgroups according to donor age: Group 1 (1^st^-2^nd^ decade, n=34), Group 2 (3^rd^-4^th^ decade, n=51), Group 3 (5^th^-6^th^ decade, n=75) and Group 4 (7^th^-8^th^ decade, n=28). Donor, recipient and transplantation characteristics were compared in the 4 groups. ANOVA test and Kruskal-Wallis test were used for the comparison of continuous and categorical variables. Kaplan-Meier test was adopted for survival analysis: log-rank test was used for comparison among the groups’ survival rates.

## Results

**Figure 1:**
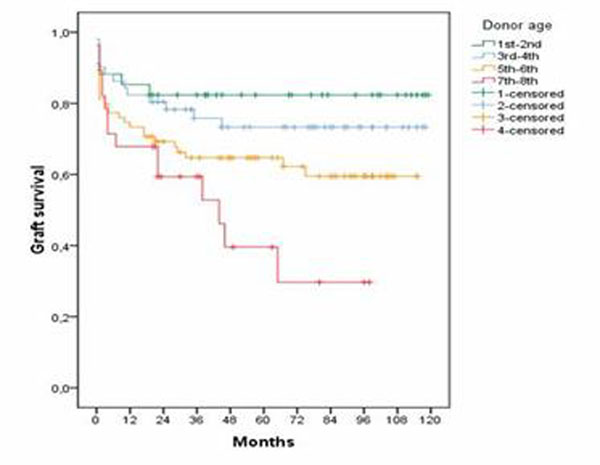
Graft survival in the 4 groups.

As expected, donor age, percentage of cerebrovascular deaths, BMI and DRI resulted higher in the last group. The male gender was prevalent in the 1^st^ Group, while macrovescicular steatosis resulted higher in the 3^rd^ Group. Recipient and immediate post-transplant features resulted homogeneous among the groups. At survival analysis, 5-year graft survival rates were progressively worsened among the groups (82.4 *vs* 73.3 *vs* 64.7 *vs* 39.6%, respectively). At log-rank analysis, statistical significance was observed between the first 2 Groups and 4^th^ one (*p*-value 0.003 and 0.006, respectively), while a boundary statistical significance was observed between the 1^st^ and 3^rd^ Group. Figure 1.

## Conclusions

In our experience, use of < 70 year-aged donors seems to be safe, while very aged (over 70) donors give poor long-term survival rates, despite similar initial post-transplantation results. We could speculate that grafts procured by very aged donors could be easier targets of viral recurrence, late ischemia-reperfusion damage and chronic rejection. A better allocation system for these organs may be improved, preferring HCC recipients who exceed transplant criteria to HCV ones [3].
